# Supplementary material for: A comprehensive thermodynamic model for RNA binding by the Saccharomyces cerevisiae Pumilio protein PUF4
Source: Nat Commun. 2022 Aug 4;13:4522. doi: 10.1038/s41467-022-31968-z (PMC9352680; doi:10.1038/s41467-022-31968-z)
Supplement: Supplementary file 1 — Supplementary Information [file 41467_2022_31968_MOESM1_ESM.pdf]

Supplemental Information:

A comprehensive thermodynamic model for RNA binding by the *Saccharomyces cerevisiae* Pumilio protein PUF4

Christoph Sadée, Lauren D. Hagler, Winston R. Becker, Inga Jarmoskaite, Pavanapuresan P. Vaidyanathan, Sarah K. Denny, William J. Greenleaf, and Daniel Herschlag

Supplemental Figures and Tables:

```
PUF4 TNSNSAEKQRK-IEESSRFADAVLDQYIGSIHSLCKDQHGCRFLQKQLDILGSKAADAIFEETKDYTVEL 69
PUF3 HRSPLLEQLRNSSSDKNSNSNMSLKDIFGHSLEFCKDQHGSRFIQRELATSPASEKEVIFNEIRDDAIEL 70
PUM2 GRSRLLEDFRN-----NRFPNLQLRDLIGHIVEFSQDQHGSRFIQQKLERATPAERQMVFNEILQAAYQL 65

PUF4 MTDSFGNYLIQKLLEEVTTEQRIVLTKISSPHFVEISLNPHGTRALQKLIECIKTDEEA--QIVVDSLRP 137
PUF3 SNDVFGNYVIQKFFEFGSKIQKNTLVDQFKGNMKQLSLQMYACRVIQKALEYIDSNQRI--ELVLE-LSD 137
PUM2 MTDVFGNYVIQKFFEFGSLDQKLALATRIRGHVLPLALQMYGCRVIQKALESISSDQQVISEMVKE-LDG 134

PUF4 YTVQLSKDLNGNHVIQKCLQRLKPENFQFIFDAISDSCIDIATHRHGCCVLQRCLDHGTTEQCDNLCDKL 207
PUF3 SVLQMIKDQNGNHVIQKAIETIPIEKLPFILSSLTGHIYHLSTHSYGCRVIQRLLEFGSSEDQESILNEL 207
PUM2 HVLKCVKDQNGNHVVQKCIECVQPQSLQFIIDAFKGQVFVLSTHPYGCRVIQRILEHCTAEQTLPILEEL 204

PUF4 LALVDKLTLDPFGNYVVQYIITKEAEKN--KYDYTHKIVHLLKPRAIELSIHKFGSNVIEKILKTAIVSE 275
PUF3 KDFIPYLIQDQYGNYVIQYVLQQDQFTNKEMVDIKQEIIETVANNVVEYSKHKFASNVVEKSILYGSKNQ 277
PUM2 HQHTEQLVQDQYGNYVIQHVLEHGRPED------KSKIVSEIRGKVLALSQHKFASNVVEKCVTHASRAE 268

PUF4 PMIL--EIL--NNG------GETGIQSLLNDSYGNYVLQTALDISHKQNDYLYKRLSEIVAPLLVGPIRN 335
PUF3 KDLIISKILPRDKNHALNLEDDSPMILMIKDQFANYVIQKLVNVSEGEGK---KLIVIAIRAYLDKLNKS 344
PUM2 RALLIDEVCCQNDG------PHSALYTMMKDQYANYVVQKMIDMAEPAQR---KIIMHKIRPHITTL-RK 328

PUF4 TPHGKRIIGMLHLDS 350
PUF3 NSLGNRHLASVEKLA 359
PUM2 YTYGKHILAKLEKYY 343
```

Supplementary Fig. 1 BLAST sequence alignment of *S. cerevisiae* PUF4, *S. cerevisiae* PUF3, and human PUM2. Full sequence alignment of the Pumilio homology domain of *S. cerevisiae* PUF4 (top row), *S. cerevisiae* PUF3 (middle row), and human PUM2 (bottom row) using blastp (https://blast.ncbi.nlm.nih.gov/Blast.cgi). The amino acid reference positions are shown on the right. Grey boxes represent the amino acids that contact the nucleic acids in the eight Pumilio repeats.

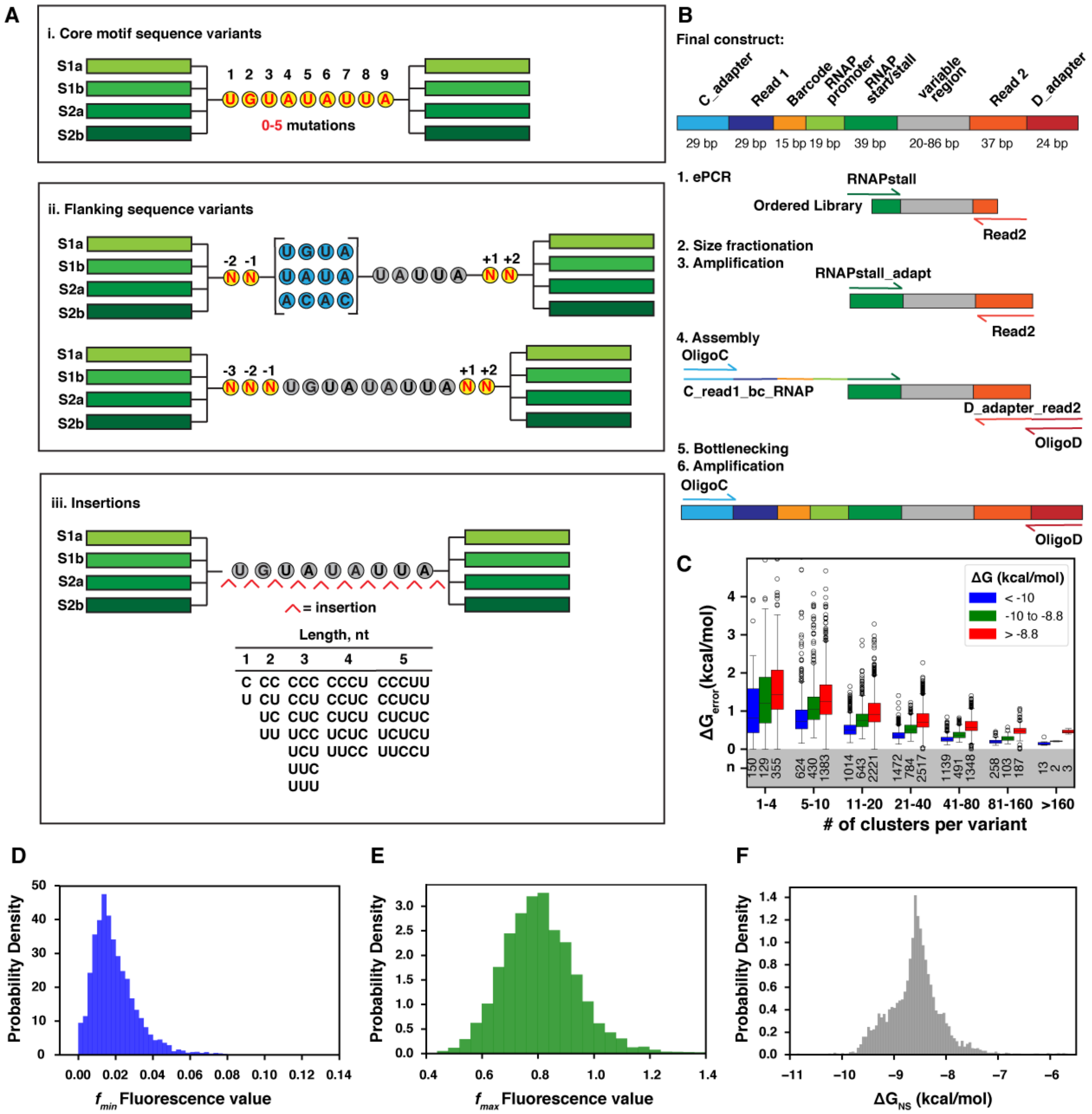
A
i. Core motif sequence variants
S1a
S1b
S2a
S2b
1 2 3 4 5 6 7 8 9
U G U A U A U U A
0-5 mutations
ii. Flanking sequence variants
-2 -1
N N
U G U A
U A U A
A C A C
U A U U A
+1 +2
N N
-3 -2 -1
N N N U G U A U A U U A N N
iii. Insertions
U G U A U A U U A
= insertion
Length, nt
1 2 3 4 5
C CC CCC CCCU CCCUU
U CU CCU CCUC CCUCU
UC CUC CUCU CUCUC
UU UCC UCUC UCUCU
UCU UUCC UUCCU
UUC
UUU
B
Final construct:
C_adapter
Read 1
Barcode
RNAP promoter
RNAP start/stall
variable region
Read 2
D_adapter
29 bp 29 bp 15 bp 19 bp 39 bp 20-86 bp 37 bp 24 bp
1. ePCR
RNAPstall
Ordered Library
Read2
2. Size fractionation
3. Amplification
RNAPstall_adapt
Read2
4. Assembly
OligoC
C_read1_bc_RNAP
D_adapter_read2
OligoD
5. Bottlenecking
6. Amplification
OligoC
OligoD
C
ΔG (kcal/mol)
< -10
-10 to -8.8
> -8.8
ΔGerror (kcal/mol)
n
150 129 355
624 430 1383
1014 643 2221
1472 784 2517
1139 491 1348
258 103 187
13 2 3
1-4 5-10 11-20 21-40 41-80 81-160 >160
# of clusters per variant
D
Probability Density
fmin Fluorescence value
E
Probability Density
fmax Fluorescence value
F
Probability Density
ΔGNS (kcal/mol)

Supplementary Fig. 2 Designed RNA library preparation and experiments (Related to Fig. 2a). **a** Designed RNA libraries used in this study. The RNA library was designed based on three Pumilio consensus sequences (PUF4, PUM2, and PUF5). For simplicity, only the variants relative to the PUF4 consensus motif are shown. A full list of all sequences and mutants in all RNA libraries studied herein can be found in Supplementary Table 1 and in ref. 1. Each variant in the designed library was inserted in 2-4 scaffolds (S1a-S2b, Fig. 2a). Variations relative to the consensus motif can be grouped into three categories. i. Motif mutations, where 1-5 bases were changed. A maximum of two bases were mutated in the conserved 5' core UGUA to avoid a predominance of unmeasurable low affinity binders. ii. Flanking sequence variants, where bases upstream and downstream of the consensus are varied to assess their binding effects. iii. Insertions, where 0-5 C or U bases were inserted between the residues of the consensus motif. **b** The DNA array library was prepared as described by ref. 1. See "Methods" for detailed sequence information and description of each step. **c** The number of clusters per variant from the chip used in low-salt measurements and affinity correlate with measurement certainty. Box plots are grouped into high, medium, and low affinity binders, with the lowest affinity binder group representing RNAs with <15% bound RNA at highest protein concentration. The orange line represents the median, the box the interquartile range, whiskers are max and min values and circles show outliers. ΔG_{error} was calculated from the 95% confidence interval as described in the "Methods." **d-f** Distribution of initial fit parameters for variant-independent parameters. Histogram of high affinity variants with >85% RNA bound at highest concentration based on initial per variant K_D and other criteria as described in "Methods". (D) f_{min} (E) f_{max} (F) ΔG_{NS}.

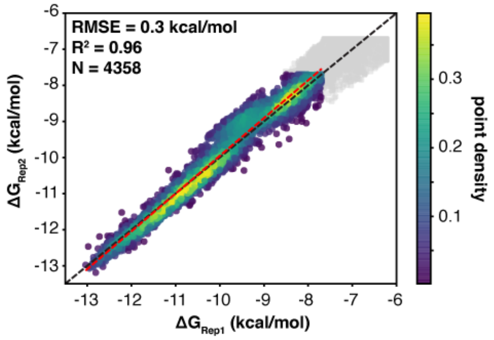

Supplementary Fig. 3 Comparison of replicate data. Two technical replicates of PUF4 binding measurements under the high salt condition (2 mM $MgCl_2$, 100 mM KOAc) were fit as described in "Methods". Note these are higher salt conditions than used in the main text; lower salt was used to enhance binding and thus the number of RNAs that gave measurable K_D values]. The transparent region (above -7.7 kcal/mol) corresponds to ΔG values that cannot be distinguished from background with high confidence. N is the number of values in the high confidence range. The black dashed line represents a correlation line (x = y). The red dashed line is the best fit line with a slope of 1.05 and intercept of 0.51 kcal/mol.

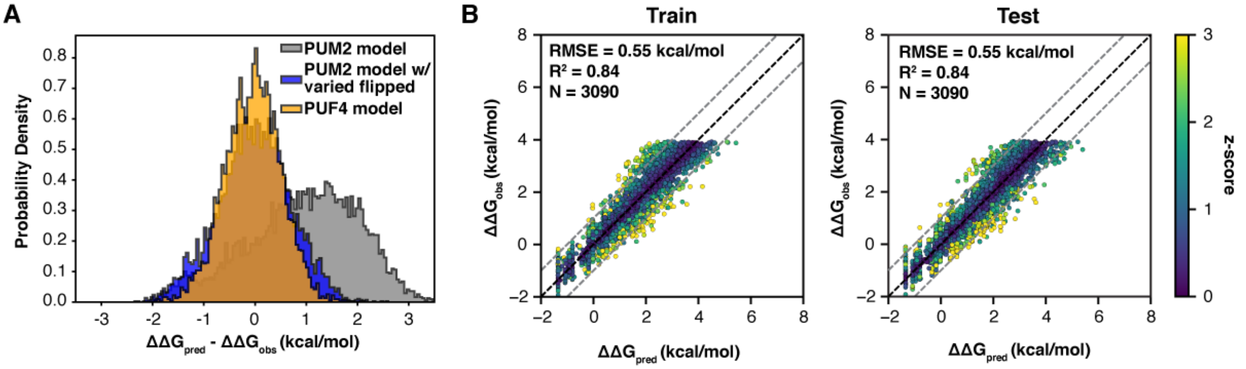

Supplementary Fig. 4 Comparison of predictive models for PUM2/PUF4 specificity (Related to Fig. 3). **a** Distribution of differences between model predictions and observed affinities. The grey plot corresponds to the data fit to the PUM2 model as described in ref. 1 (Fig. 3b). The blue plot corresponds to the data fit to the PUM2 model with all flipping parameters optimized as described in "Methods" (Fig. 3C). The orange plot corresponds to the PUF4 model derived herein (Fig. 3d). **b** To test the model for overfitting, data were initially randomly and split into equal-sized training and test datasets. The model was optimized on the training dataset and the resulting parameters were used to predict the test data. RMSE and R^2 were used to assess goodness of fit, and these parameters were equal for the two datasets.

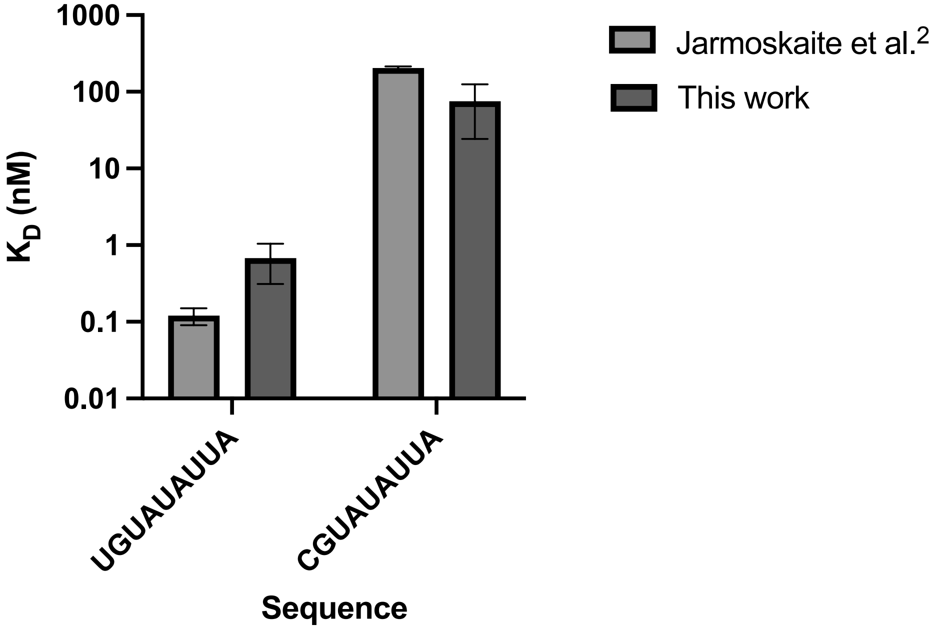

Supplementary Fig. 5. Comparison of RNA-MaP PUF4 affinities to literature values. Published literature affinities for PUF4 binding to RNAs measured at 25 °C, following the criteria for reliable measuring binding affinities outlined in ref. 2 (i.e., sufficient equilibration time and [RNA] varied and < K_D), compared to the binding of similar RNA variants from this work. In ref. 2, bars depict the average K_D and standard error from 5 independent experiments. In this work, bars represent the median K_D value and 95% confidence interval of the mean from n = 346 and n = 2 replicate clusters. Other literature values for PUF4[3,4] are not directly comparable because they were measured at different temperatures, their equilibration times were not long enough to ensure equilibration, or without varying the [RNA] (see ref. 2). On- and off-chip comparisons have been made for five other proteins with a total of 49 comparisons in addition to the two reported herein, and all show good agreement.[1,5-8]

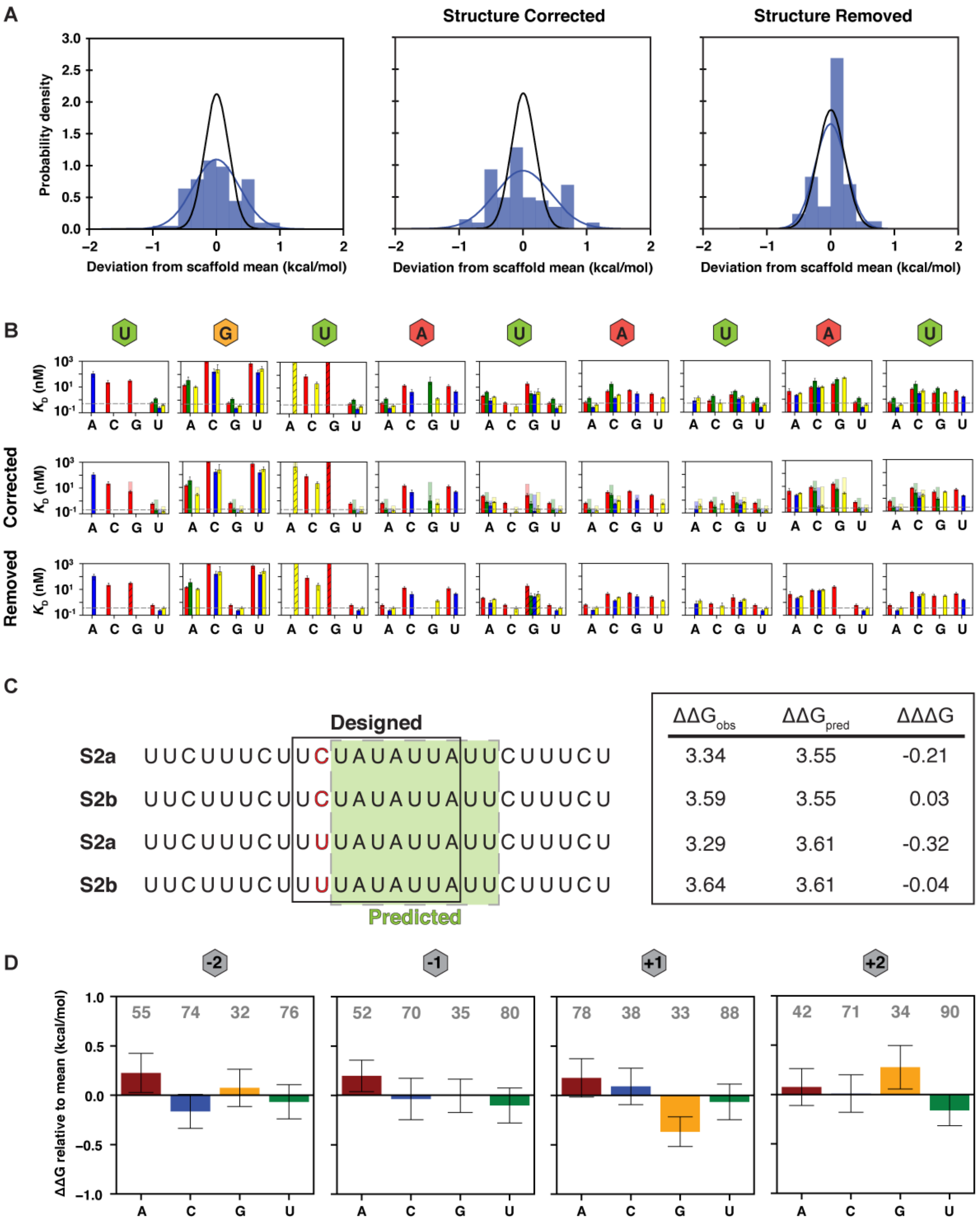
A
Structure Corrected
Structure Removed
Probability density
Deviation from scaffold mean (kcal/mol)
B
U G U A U A U A U
Corrected
Removed
K_D (nM)
A C G U
C
Designed
Predicted
S2a UUCUUUCUUCUAUAUUAUUCUUUCU
S2b UUCUUUCUUCUAUAUUAUUCUUUCU
S2a UUCUUUCUUUUAUAUUAUUCUUUCU
S2b UUCUUUCUUUUAUAUUAUUCUUUCU
ΔΔG$_{obs}$ ΔΔG$_{pred}$ ΔΔΔG
3.34 3.55 -0.21
3.59 3.55 0.03
3.29 3.61 -0.32
3.64 3.61 -0.04
D
-2 -1 +1 +2
55 74 32 76
52 70 35 80
78 38 33 88
42 71 34 90
ΔΔG relative to mean (kcal/mol)
A C G U

Supplementary Fig. 6. Analysis of single-mutant variants binding to PUF4. a The effects of scaffold differences were assessed by determining the deviation of the individual scaffold affinities from scaffold mean for that variant. The deviation was calculated for each binding site variant (single mutant and the PUF4 consensus UGUAUAUUA). The distribution of these deviations (blue line) was compared to the distribution expected from experimental error (black line). Distributions were compared without structure correction (left). Inter-scaffold variation is larger than that predicted by experimental error (blue *vs.* black lines). Correcting for structure effects using ViennaRNA fold did not resolve the deviation (center). Removing structured variants with cutoff $\Delta G_{fold} < -0.5$ (as described in "Methods") lowered inter-scaffold variation within experimental error (right). **b** PUF4 affinities for UGUAUAUUA single mutants before accounting for structure (top), with structure correction (middle), and after structured variants were removed (bottom). Scaffolds are depicted with colored bars (red = S1a, green = S1b, blue = S2a, yellow = S2b). Hashed lines indicate variants not satisfying all of the quality control cutoffs, but were included because they were the only single mutant variant at that position. Error bars indicate weighted replicate errors. **c** Assessment of alternative binding registers. Alternative registers were predicted based on the register with the highest binding affinity. PUF4 predominantly bound in designed registers with minor exception for single mutants at position two, deviating no more than 0.25 kcal/mol and within experimental error. **d** Flanking sequence effects on PUF4 binding. Four bases, two downstream and two upstream of the PUF4 consensus were randomized (see also Supplementary Fig. 2) and embedded into different scaffolds (n = 256). Effects were assessed by taking the average affinity of all variants with a given base at each position. Error bars indicate the 95% CI of the mean. The number of variants for each is shown above the bar. The values were subtracted from the average of all combined variants to assess deviation from mean. Deviations were within 0.5 kcal/mol with the most significant increase at position +1 for guanine. Hence, an additional base was added to the PUF4 consensus to account for the additional energy term in global fitting, yielding this 10-mer UGUAUAUUAU. U at position 10 was chosen over G due to its large representation in the library. Structured variants with $\Delta G_{fold} < -0.5$ were removed for this analysis.

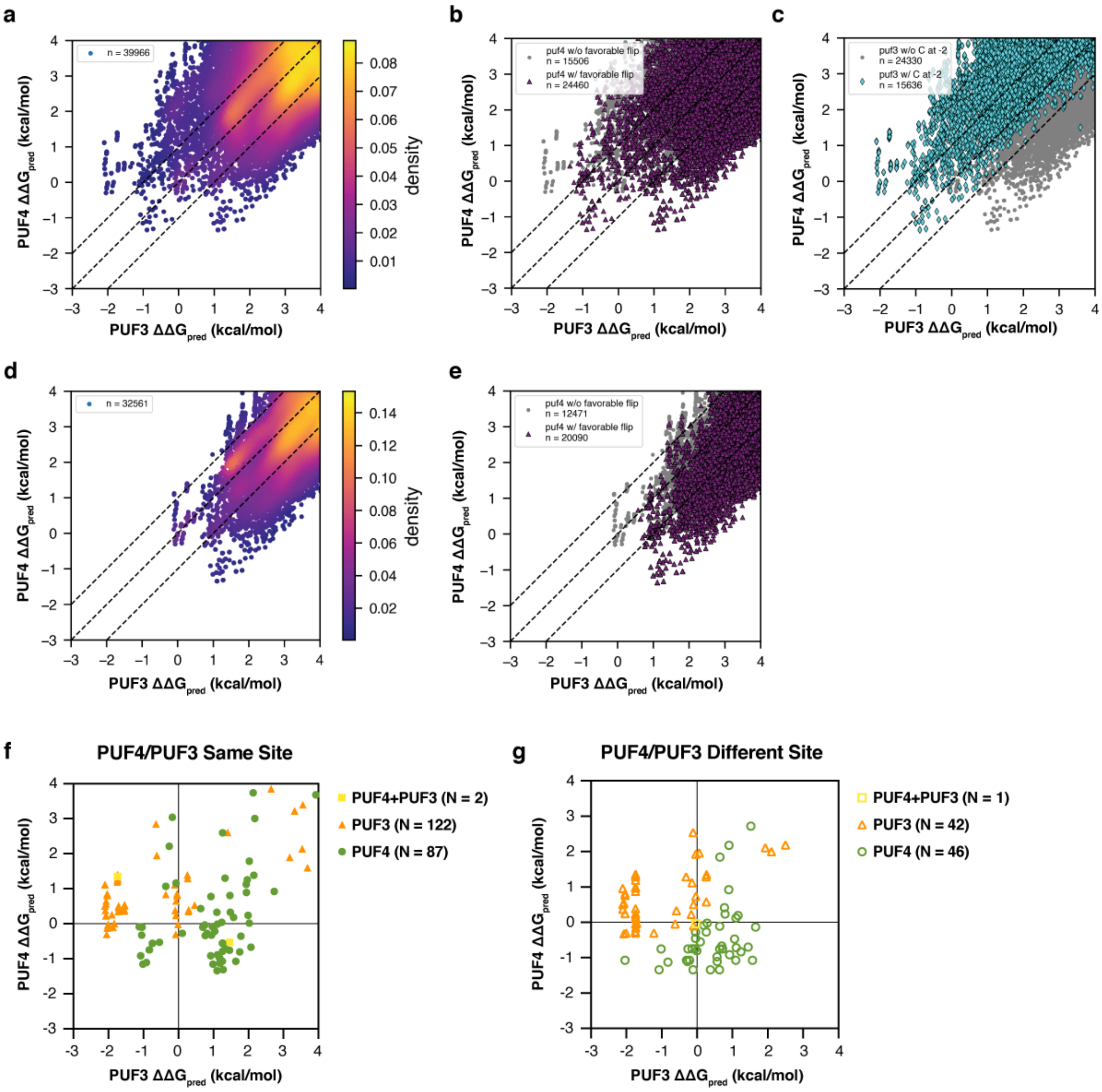
a
n = 39966
density
PUF4 ΔΔG_pred (kcal/mol)
PUF3 ΔΔG_pred (kcal/mol)
b
puf4 w/o favorable flip
n = 15506
puf4 w/ favorable flip
n = 24460
c
puf3 w/o C at -2
n = 24330
puf3 w/ C at -2
n = 15636
d
n = 32561
e
puf4 w/o favorable flip
n = 12471
puf4 w/ favorable flip
n = 20090
f
PUF4/PUF3 Same Site
PUF4+PUF3 (N = 2)
PUF3 (N = 122)
PUF4 (N = 87)
g
PUF4/PUF3 Different Site
PUF4+PUF3 (N = 1)
PUF3 (N = 42)
PUF4 (N = 46)

Supplementary Fig. 7. Transcriptome-wide analysis of PUF4 and PUF3 binding sites in *S. cerevisiae* 3'UTRs (Related to Fig. 6). a-c PUF4/PUF3 affinity–specificity landscape for 13 nt windows of *S. cerevisiae* 3'UTRs in the Saccharomyces genome database. See also "Methods". $\Delta\Delta G_{pred}$ is the ensemble binding energy for each window relative to the consensus sequence for each RBP (PUF4 = UGUAUAUUA, PUF3 = CNUGUAUAUA), obtained using the appropriate mathematical model (see Table 1 and Supplementary Table 2); all sites with $\Delta\Delta G_{ensemble} < 4$ kcal/mol are included. **a** is colored by point density; **b** compares binding sites where the PUF4 model utilizes the 6/7 flipping term (purple triangle); **c** compares binding sites where the PUF3 model utilizes the C at position -2 term (cyan diamond). **d,e** PUF4/PUF3 affinity–specificity landscape for 11 nt windows of *S. cerevisiae* 3'UTRs in the Saccharomyces genome database as in **a,b**, with the consensus sequence for PUF3 being UGUAUAUA. See also "Methods". **f,g** PUF4/PUF3 affinity–specificity landscapes for the highest affinity binding site for previously identified PUF3 and PUF4 3'UTR targets[9]. In **f**, the highest affinity site is the same for PUF3 and PUF4, and in **g**, the highest affinity sites are different for PUF3 and PUF4. PUF3 targets are depicted by an orange triangle; targets that bind PUF3 and PUF4 with a yellow square, and PUF4 targets with a green circle. N is the number of known targets for each.

Supplementary Table 1: Flipped Parameters for PUM2 model with optimized flips (Related to Fig. 3c)

Term II	$\Delta\Delta G_f^Y$ (kcal/mol)				
	Y =				
Flipped Residue Position *f* =	**A**	**C**	**U**	**G**	**NN**[a]
**3/4**	2.00	1.12	2.24	1.02	2.50
**4/5**	2.00	0.30	1.23	1.71	2.50
**5/6**	2.00	0.47	1.32	1.57	2.38
**6/7**	0.03	-0.82	-0.82	-0.07	1.88
[a]2- nt flip of any sequence					

Supplementary Table 2. Parameters used for PUF3 Model (Related to Fig. 6e,f and Supplementary Fig. 6)

**PUF3 Thermodynamic values**					
Term I	$\Delta\Delta G_b^X$ (kcal/mol)				
	X =				
Bound Residue Position *b* =	**A**	**C**	**U**	**G**	
**-2**	0.00	-2.00	0.00	0.00	
**-1**	0.0	0.00	0.00	0.00	
**1**	3.08	2.91	0.00	3.04	
**2**	1.93	3.14	3.14	0.00	
**3**	2.39	2.49	0.00	2.92	
**4**	0.00	1.92	1.46	1.71	
**5**	-0.03	0.17	0.00	0.79	
**6**	0.00	1.83	1.49	1.82	
**7**	1.55	1.78	0.00	1.59	
**8**	0.00	1.57	1.01	1.52	
**9**	0.30	0.29	0.00	-0.07	
Term II	$\Delta\Delta G_f^Y$ (kcal/mol)				
	Y =				
Flipped Residue Position *f* =	**A**	**C**	**U**	**G**	**NN**[a]
**3/4**	>2.00	1.79	1.41	>1.5	>2.50[b]
**4/5**	>2.00	>3.00	>2.50	>2.50	>2.50
**5/6**	1.22	1.05	0.81	1.57	2.18
**6/7**	>2.00	1.77	>2.02	>2.0	2.04
[a]2- nt flip of any sequence					
[b] > indicates a lower limit					

Supplementary Table 3. Sequences for Oligonucleotides used in Library Assembly (related to Supplementary Fig. 2b; reproduced from reference 2).

	Name	Sequence (sense strand)
Components of the RNA array construct	C_adaptor	AATGATACGGCGACCACCGAGATCTACAC
	Read1	TCTTTCCCTACACGACGCTCTTCCGATCT
	Barcode*	NNNNNNNNNNNNNNN
	RNAP promoter	TTTATGCTATAATTATTTC
	RNAP start/stall sequence**	ATGTAGTAAGGAGGTTGTATGGAAGACGTTCCTGGATCC
	Read2’	AGATCGGAAGAGCGGTTCAGCAGGAATGCCGAGACCG
	D’_adaptor	ATCTCGTATGCCGTCTTCTGCTTG
Primers used in library preparation	RNAPstall	GTAAGGAGGTTGTATGGAAGACGTTCCTGGATCC
	Read2	CGGCATTCCTGCTGAACCGCTCTTCCGATCT
	RNAPstall_adapt	GTAAGGAGGTTGTATGGAAGACGTTCCTGGAT
	C_read1_bc_RNAP	AATGATACGGCGACCACCGAGATCTACACTCTTTCCCTACACGACGCTCTTCCGATCTNNNNNNNNNNNNNNNTTTATGCTATAATTATTTCATGTAGTAAGGAGGTTGTATGGAAGACGTTCCTGGAT
	D_read2	CAAGCAGAAGACGGCATACGAGATCGGTCTCGGCATTCCTGCTGAACCGCTCTTCCGATCT
	OligoC	AATGATACGGCGACCACCGAGATCTACAC
	OligoD	CAAGCAGAAGACGGCATACGAGAT
DNA oligonucleotides used in RNA array preparation	Fiducial_chip***	AATGATACGGCGACCACCGAGATCTACACTCTTTCCCTACACGACGCTCTTCCGATCTCTTGGGTCCACAGGACACTCGTTGCTTTCCAGATCGGAAGAGCGGTTCAGCAGGAATGCCGAGACCGATCTCGTATGCCGTCTTCTGCTTG
	Fudicial_flow	/5TYE563/GGAAAGCAACGAGTGTCCTGTGGACCCAAG
	Fluorecent_stall’	GGATCCAGGAACGTCTTCCATACAACCTCCTTACTACAT/3AlexF647N/
	Biotin_D_Read2	/5BiosG/CAAGCAGAAGACGGCATACGAGATCGGTCTCGGCATTCCTGCTGAACCGCTCTTCCGATCT
	Dark_stall’	GGATCCAGGAACGTCTTCCATACAACCTCCTTACTACAT
	Dark_read2	CGGCATTCCTGCTGAACCGCTCTTCCGATCT

*Randomized 15mer region
**Underlined ‘C’ indicates the RNA polymerase stall site
***Corresponds to 5’—[C_adaptor][Read1] CTT GGG TCC ACA GGA CAC TCG TTG CTT TCC [Read2’][D’_adaptor]

References:

1. Jarmoskaite, I. *et al.* A quantitative and predictive model for RNA binding by human Pumilio proteins. *Mol. Cell* **74**, 966-981 (2019).
2. Jarmoskaite, I., Alsadhan, I., Vaidyanathan, P. P. & Herschlag, D. How to measure and evaluate binding affinities. *Elife* **9**, 1–34 (2020).
3. Miller, M. T., Higgin, J. J. & Tanaka Hall, T. M. Basis of altered RNA-binding specificity by PUF proteins revealed by crystal structures of yeast Puf4p. *Nat. Struct. Mol. Biol.* **15**, 397–402 (2008).
4. Hook, Brad A. *et al.* Two yeast PUF proteins negatively regulate a single mRNA. *J. Biol. Chem.* **282**, 15430-15438 (2007).
5. Buenrostro, J. D. *et al.* Quantitative analysis of RNA-protein interactions on a massively parallel array reveals biophysical and evolutionary landscapes. *Nat. Biotechnol.* **32**, 562–568 (2014).
6. She, R. *et al.* Comprehensive and quantitative mapping of RNA-protein interactions across a transcribed eukaryotic genome. *Proc. Natl. Acad. Sci. U. S. A.* **114**, 3619–3624 (2017).
7. Tome, J. M. *et al.* Comprehensive analysis of RNA-protein interactions by high-throughput sequencing-RNA affinity profiling. *Nat. Methods* **11**, 683–688 (2014).
8. Nutiu, R. et al. Direct measurement of DNA affinity landscapes on a high-throughput sequencing instrument. *Nat. Biotech.* **29**, 659-664 (2011).
9. Gerber, A. P., Herschlag, D. & Brown, P. O. Extensive association of functionally and cytotopically related mRNAs with Puf family RNA-binding proteins in yeast. *PLoS Biol.* **2**, 342-354 (2004).
