# Supplementary material for: A comprehensive thermodynamic model for RNA binding by the Saccharomyces cerevisiae Pumilio protein PUF4
Source: Nat Commun. 2022 Aug 4;13:4522. doi: 10.1038/s41467-022-31968-z (PMC9352680; doi:10.1038/s41467-022-31968-z)
Supplement: Supplementary file 3 — Reporting Summary [file 41467_2022_31968_MOESM3_ESM.pdf]

nature portfolio

Corresponding author(s): Dan Herschlag

Last updated by author(s): Jun 13, 2022

Reporting Summary

Nature Portfolio wishes to improve the reproducibility of the work that we publish. This form provides structure for consistency and transparency in reporting. For further information on Nature Portfolio policies, see our Editorial Policies and the Editorial Policy Checklist.

Statistics

For all statistical analyses, confirm that the following items are present in the figure legend, table legend, main text, or Methods section.

n/a	Confirmed	
☐	☒	The exact sample size (*n*) for each experimental group/condition, given as a discrete number and unit of measurement
☒	☐	A statement on whether measurements were taken from distinct samples or whether the same sample was measured repeatedly
☒	☐	The statistical test(s) used AND whether they are one- or two-sided<br>*Only common tests should be described solely by name; describe more complex techniques in the Methods section.*
☒	☐	A description of all covariates tested
☐	☒	A description of any assumptions or corrections, such as tests of normality and adjustment for multiple comparisons
☐	☒	A full description of the statistical parameters including central tendency (e.g. means) or other basic estimates (e.g. regression coefficient) AND variation (e.g. standard deviation) or associated estimates of uncertainty (e.g. confidence intervals)
☒	☐	For null hypothesis testing, the test statistic (e.g. *F*, *t*, *r*) with confidence intervals, effect sizes, degrees of freedom and *P* value noted<br>*Give P values as exact values whenever suitable.*
☒	☐	For Bayesian analysis, information on the choice of priors and Markov chain Monte Carlo settings
☒	☐	For hierarchical and complex designs, identification of the appropriate level for tests and full reporting of outcomes
☐	☒	Estimates of effect sizes (e.g. Cohen's *d*, Pearson's *r*), indicating how they were calculated

Our web collection on statistics for biologists contains articles on many of the points above.

Software and code

Policy information about availability of computer code

Data collection	Matlab (MathWorks Inc., 2010b) was used to capture images on the custom Illumina sequencing platform of RNA-MaP https://www.nature.com/articles/nbt.2880#Sec28. Matlab was used to analyze image and detect clusters of RNA-protein interactions. Matlab was used for determine the fluorescence of each clusters. Off-chip analysis of PUF4 fraction active were quantified using TotalLab Quant and fit with KaleidaGraph 4.1.
Data analysis	Matlab (MathWorks Inc., 2010b) was used to calculate the normalized fluorescence from images of the custom Illumina sequencing platform of RNA-MaP. Python 2.7 was used to derive affinity parameters using the RNA-MaP package array-fitting-tool https://github.com/GreenleafLab/array_fitting_tools. Python 2.7 was used for model development, general data analysis, and plotting (https://github.com/HerschlagLab/PUF4Model.git). Vienna RNAfold 2.4.18 was used to predict the RNA structure within python 2.7. GraphPad Prism 9 was used to plotting figures and linear fits.

For manuscripts utilizing custom algorithms or software that are central to the research but not yet described in published literature, software must be made available to editors and reviewers. We strongly encourage code deposition in a community repository (e.g. GitHub). See the Nature Portfolio guidelines for submitting code & software for further information.

Data

Policy information about availability of data

All manuscripts must include a data availability statement. This statement should provide the following information, where applicable:

- Accession codes, unique identifiers, or web links for publicly available datasets
- A description of any restrictions on data availability
- For clinical datasets or third party data, please ensure that the statement adheres to our policy

Source data for Figs 2c and 3b-d, including the thermodynamic binding data generated in this study are available in Supplementary Data. All other data supporting the findings of this study are available from the corresponding author on request.

Field-specific reporting

Please select the one below that is the best fit for your research. If you are not sure, read the appropriate sections before making your selection.

- [x] Life sciences
- [ ] Behavioural & social sciences
- [ ] Ecological, evolutionary & environmental sciences

For a reference copy of the document with all sections, see nature.com/documents/nr-reporting-summary-flat.pdf

Life sciences study design

All studies must disclose on these points even when the disclosure is negative.

Sample size	The cutoff for number of clusters per variant (≥5 clusters per variant) was determined from statistical analysis. This number of clusters (where each cluster represents ~1000 RNAs) gave the most robust statistics, minimizing the error on ΔG.
Data exclusions	Variants were included in our analysis if they met the following criteria: (1) Variants with ΔGerror < 1.0 kcal/mol, (2) Five or more clusters per variant in each experiment and replicate, and (3) observed ΔG values less than -7.69 kcal/mol, where more than 15% of RNA was bound at the highest protein concentration. These exclusions were pre-determined in our previous publication Jarmoskaite et al. 2019.
Replication	The "high" salt condition was replicated independently (i.e., new library preparation, sequenced on a different chip, independent binding measurements). All attempts to replicate were successful as shown with low error between replicates.
Randomization	Not applicable. No randomization was needed in this study because binding measurements are in vitro and quantitative.
Blinding	Not applicable. No blinding was used in this study, because quantitative measurements of binding affinity were performed and samples were treated equally.

Reporting for specific materials, systems and methods

We require information from authors about some types of materials, experimental systems and methods used in many studies. Here, indicate whether each material, system or method listed is relevant to your study. If you are not sure if a list item applies to your research, read the appropriate section before selecting a response.

Materials & experimental systems

n/a	Involved in the study	
☒	☐	Antibodies
☒	☐	Eukaryotic cell lines
☒	☐	Palaeontology and archaeology
☒	☐	Animals and other organisms
☒	☐	Human research participants
☒	☐	Clinical data
☒	☐	Dual use research of concern

Methods

n/a	Involved in the study	
☒	☐	ChIP-seq
☒	☐	Flow cytometry
☒	☐	MRI-based neuroimaging
